# Panax notoginsenoside Rb1 ameliorates Alzheimer’s disease by upregulating brain-derived neurotrophic factor and downregulating Tau protein expression

**DOI:** 10.3892/etm.2013.1215

**Published:** 2013-07-11

**Authors:** YU WANG, YU FENG, QUNYING FU, LEI LI

**Affiliations:** Department of Neurology, The First Affiliated Hospital of China Medical University, Shenyang, Liaoning 110015, P.R. China

**Keywords:** Alzheimer’s disease, Panax notoginsenoside Rb1, okadaic acid, phosphorylated Tau protein, brain-derived neurotrophic factor

## Abstract

Alzheimer’s disease (AD) is a neurodegenerative disorder and the main cause of dementia. Panax notoginsenoside Rb1 (PNRb1), which is also known as (3β,12β)-20-[(6-O-β-D-glucopyranosyl-β-D-glucopyranosyl)oxy]-12-hydroxydammar-24-en-3-yl 2-O-β-D-glucopyranosyl-β-D-glucopyranoside and is the main active component of the plant *Panax notoginseng*, is effective in treating AD. However, the mechanisms of PNRb1 remain unknown. In the present study, rat brain tissue sections were pretreated with PNRb1 and then induced by okadaic acid to establish brain slice models of AD. The results of qPCR and immunoblot analyses demonstrated that PNRb1 suppressed the protein expression of phosphorylated Tau and upregulated the expression levels of brain-derived neurotrophic factor (BDNF). These results suggest that PNRb1 is able to upregulate the protein level of BDNF and downregulate Tau protein phosphorylation in AD.

## Introduction

Alzheimer’s disease (AD), the cause of one of the most common types of dementia, is a brain disorder affecting the elderly. It is characterized by the formation of two main protein aggregates, senile plaques and neurofibrillary tangles, which are involved in a process leading to progressive neuronal degeneration and death (1). Several agents have demonstrated the ability to enhance cognition and global function in patients with AD. Advances in the understanding of AD pathogenesis have resulted in the development of numerous compounds that may modify the disease process. In addition, a wide array of anti-amyloid and neuroprotective therapeutic approaches are under investigation (2). Limiting oxidation and toxicity, reducing Tau phosphorylation and controlling inflammation may be beneficial disease-modifying strategies. Moreover, potential neuroprotective and restorative treatments, such as neurotrophins, neurotrophic factor enhancers and stem cell-related approaches, are also under investigation (3).

Neural stem cells (NSCs) may offer an alternative source for curing patients with AD. Epidermal neural crest stem cells (EPI-NCSCs) are capable of differentiating into neurons, astrocytes and oligodendrocytes. Transplantation of EPI-NCSCs into the hippocampus was demonstrated to result in the generation of cholinergic neurons that were able to cure memory impairment in a rat model of AD (4). NSC transplantation represents an unexplored approach for treating neurodegenerative disorders associated with cognitive decline, such as AD. A previous study demonstrated that NSCs ameliorated complex behavioral deficits associated with widespread AD pathology via brain-derived neurotrophic factor (BDNF) (5).

Human cellular models of AD pathogenesis would enable investigation of the candidate pathogenic mechanisms of AD, and the evaluation and development of novel therapeutic strategies. Shi *et al* demonstrated the development of AD pathologies in cortical neurons that had been generated from human induced pluripotent stem (iPS) cells derived from patients with Down syndrome (6). It was identified that cortical neurons generated from iPS and embryonic stem cells from patients with Down syndrome developed AD pathologies. These cortical neurons processed the transmembrane APP protein, resulting in secretion of the pathogenic peptide fragment amyloid-β42 (Aβ42), which formed insoluble intra- and extracellular amyloid aggregates. However, the production of Aβ peptides was blocked by a γ-secretase inhibitor. In addition, hyperphosphorylated Tau protein, a pathological hallmark of AD, was localized to cell bodies and dendrites in iPS cell-derived cortical neurons from patients with Down syndrome, recapitulating the later stages of the AD pathogenic process. Furthermore, Yahata *et al* differentiated human iPS cells into neuronal cells expressing the forebrain marker, Foxg1 and the neocortical markers, Cux1, Satb2, Ctip2 and Tbr1 (7). The iPS cell-derived neuronal cells also expressed amyloid precursor protein, as well as β- and γ-secretase components, and were capable of secreting Aβ into the conditioned media. Aβ production was inhibited by β- and γ-secretase inhibitors (GSI) and a nonsteroidal anti-inflammatory drug. These results indicated that the human iPS cell-derived neuronal cells expressed functional β- and γ-secretases involved in Aβ production. However, it remains unclear whether this approach would be transferable to human patients; additional studies are required to ensure the safety of cell transplantation into the brain. Further studies are also needed to improve the effectiveness of transplants, avoid the potential side-effects, investigate the mechanisms of AD and determine how cells may assist with the development of novel treatment agents.

A number of studies have focused on traditional medicinal plants for the development of novel therapeutic agents that lack side-effects. Medicinal herbs have long been used in Asia to treat various neurological diseases, including strokes and epilepsy (8–10). Panax notoginsenoside Rb1 (PNRb1; (3β,12β)-20-[(6-O-β-D-glucopyranosyl-β-D-glucopyranosyl)oxy]-12-hydroxydammar-24-en-3-yl 2-O-β-D-glucopyranosyl-β-D-glucopyranoside, is the main bioactive component of *Panax notoginseng,* which promotes neurotransmitter release by modulating phosphorylation of the synapsis through a cAMP-dependent protein kinase pathway (11). Notoginsenoside has the same chemical structure as ginsenoside; however, in China, these molecules are differentiated, as the former is extracted from the plant *Panax notoginseng* and the latter is extracted from the plant *Panax ginseng. Panax notoginseng* increases memory and cognitive functions (12), and has been effectively used to protect neurons and promote functional rehabilitation in patients following cerebral hemorrhage (13). A previous study has shown that *Panax notoginseng* saponins (PNS; key components of *Panax notoginseng*) protect against the formation of pathological lesions of cholinergic neurons in a rat model of AD (14). Modern pharmacological studies have demonstrated that PNS ameliorates and protects against neuropathological impairment. Furthermore, PNS remarkably improves spatial learning and memory in rats with AD (15). Moreover, there are four main components of PNS: Panax notoginsenoside R1 and ginsenosides Rg1, Rd and Rb1. Ginsenoside Rg1 (the same as Panax notoginsenoside R1) upregulated brain-derived neurotrophic factor (BDNF) expression and inhibited Tau protein phosphorylation in the brain slices of a rat model of AD (16). However, the proportions of PNRb1 and Panex ginsenoside Rg1 are 30–40 and 25–35%, respectively. Therefore, the present study explored whether PNRb1 has similar functions to ginsenoside Rg1 in the treatment of AD.

## Materials and methods

### Wistar rats

Experiments were performed at the Biomedicine Experimental Center, College of Medicine (The First Affiliated Hospital of China Medical University, Shenyang, China) from July 2011 to May 2012. The experimental animals were healthy male Wistar rats (age, 5 weeks; weight, 100–150 g) supplied by the Experimental Animal Center, College of Medicine (The First Affiliated Hospital of China Medical University). All animal experiments were conducted in strict accordance with the National Institutes of Health guidelines (2011, Eighth Edition) regarding humane treatment for the care and use of laboratory animals, and were reviewed and approved by the Animal Studies Committee of The First Affiliated Hospital of China Medical University.

### Traditional Chinese medicine

PNRb1, one of the biologically active ingredients of *Panax notoginseng* (molecular formula, C_54_H_92_O_23_; molecular weight, 1,109.31), was purchased from Nanjing Zelang Medical Technological Co., Ltd. (Nanjing, China) and demonstrated a purity of ≥98% (measured by high performance liquid chromatography). In accordance with a previous method (17), brain slices from a rat model of AD were pretreated with artificial cerebrospinal fluid containing 60, 120 and 240 μM PNRb1 as described below.

### Preparation of the AD rat models

In accordance with a previous method (16), rats were anesthetized with 6% chloral hydrate (400 mg/kg; Nanfang hospital, Guangzhou, China), decapitated within 1 min and the brain was placed in buffer solution with 150 mM NaCl, 2 mM CaCl_2_, 1.2 mM MgSO_4_, 0.5 mM KH_2_PO_4_, 1.5 mM K_2_HPO_4_ and 10 mM glucose (pH 7.4), for 5 min at 4°C. Fascia on the brain and unrelated tissues were removed. Treated brain tissues were fixed on a microtome and sliced into 400-μm-thick sections, each of which contained the cortex and the hippocampus. Brain slices with low light levels were placed in 6-well plates containing artificial cerebrospinal fluid (100 mm NaCl, 20 mm NaHCO_3_, 2.5 mm KCl, 1 mm NaH_2_PO4, 1 mm MgCl_2_, 10 mm glucose). Mixed gas (95% O_2_ and 5% CO_2_) was continuously added to the artificial cerebrospinal fluid at 35°C. The brain slices were randomly assigned to the blank control group, the model group and three PNRb1 groups (n=10 per group). After 1 h of incubation, PNRb1 (dissolved in analytical grade methanol) was slowly injected using a microsyringe to the PNRb1 group slices at concentrations of 60, 120 and 240 μM. After 2 h of pretreatment, 1 μM okadaic acid (Sigma, St. Louis, MO, USA), which was dissolved in dimethyl sulfoxide, was added to the model and PNRb1 groups for 4 h for model induction. The blank control group was not administered okadaic acid or PNRb1.

### Extraction of RNA and quantification of BDNF and Tau mRNA

Total RNA was isolated from brain cells using QIAshredder and RNeasy mini kits (Qiagen, Inc., Chatsworth, CA, USA). An initial strand of cDNA was synthesized from 500 ng RNA extract, in a volume of 20 μl, using AMV reverse transcriptase XL (Takara Biotechnology Co., Ltd., Dalian, China) and by priming with random 9-mers, at 42°C for 10 min. The cDNA strand was stored at 20°C until use. The mRNA levels of BDNF and Tau were evaluated by qPCR. PCR was performed in an ABI Prism 7900 sequence detector (Applied Biosystems Inc., Foster City, CA, USA) in a final volume of 20 μl. The PCR mixture contained 10 mM Tris-HCl buffer (pH 8.3), 50 mM KCl, 1.5 mM MgCl_2_, 0.2 mM dNTP mixture, 0.5 units Ampli Taq gold enzyme (Applied Biosystems Inc.) and 0.2 M primers. The primer and probe sequences for gene amplification were as follows: BDNF, 5′-GACTCT GGAGAGCGTGAATG-3′ and 5′-CACTCACTAATACTGTCACA-3′; Tau, 5′-GACAAAAAAGCCAAGGGGGC-3′ and 5′-AGGGACGGGGTGCGGGAGCG-3′; and glyceraldehyde 3-phosphate dehydrogenase (GAPDH), 5′-CCCTTCATTGAC CTCAACTAC-3′ and 5′-CCACCTTCTTGATGTCATCAT-3′. GAPDH was used as the internal control. The Ampli Taq gold enzyme was activated by heating for 10 min at 95°C, and all genes were amplified by 50 cycles of heating for 15 sec at 95°C, followed by 1 min at 60°C.

For construction of the standard curves of positive controls, the total RNA of the primary astrocytes was reverse transcribed into cDNA and serially diluted in water in five or six log steps to afford four-fold serial dilutions of cDNA from 100 ng to 100 pg. These cDNA serial dilutions were stored at −20°C. The coefficient of linear regression for each standard curve was calculated, then the cycle threshold value of a sample was substituted into the formula for each standard curve and the relative concentration of BDNF and Tau or GAPDH was calculated. To normalize differences in the volume of total RNA added to each reaction mixture, GAPDH was used as an endogenous control. Data represent the average expression of target genes relative to the expression of GAPDH, from three independent cultures.

### Immunoblot analysis

Rat brains were lysed in an ice-cold buffer containing 50 mM Tris-HCl (pH 7.4), 150 mM NaCl, 1% (v/v) NP-40, 5 mM EDTA, 5% (v/v) glycerol, 10 μg/ml leupeptin, 10 μg/ml aprotonin, 1 mmol/l phenylmethylsulfonyl fluoride and 1 mM Na_3_VO_4_, using a polytron, and the lysates were then sonicated. The samples were diluted in water (1:4) and their protein concentrations were determined using the Bradford method with affinity-purified bovine serum albumin (Sigma) as the standard. Samples of 10 g were dissolved in Laemmli sample buffer (Bio-Rad, Hercules, CA, USA), separated on 12% acrylamide gel and transferred to polyvinylidene difluoride (PVDF) membranes. Subsequently, the blots were blocked with normal goat serum antibody, incubated in rabbit anti-rat phosphorylated Tau protein and BDNF polyclonal antibody (1:1,000 and 1:600, respectively; Boster, Wuhan, China) at 4°C overnight, then washed in phosphate-buffered saline with 0.1% Triton X-100, three times for 15 min each. As an internal control to determine whether equal quantities of protein had been loaded onto the gel, the PVDF membranes were stripped and re-probed with antitubulin (T5168; Sigma). Blots were then incubated with goat anti-rabbit antibody conjugated to horseradish peroxidase (Sigma) or mouse anti-mouse antibody conjugated to horseradish peroxidase. Immunoreactive bands were visualized by enhanced chemiluminescence (ECLplus kit; GE Healthcare Life Sciences, Shanghai, China) and quantified by densitometry with ImageJ software, version 1.45 (National Institutes of Health, Bethesda, MD, USA) according to the manufacturer’s instructions.

### Statistical analysis

The association between PNRb1 concentration and Tau or BDNF protein levels in the different groups was compared by one-way analysis of variance, followed by the post hoc test of Fisher’s protected least significant difference. Spearman’s rank correlation coefficient was used to identify the strength of the correlation between the relative expression levels of Tau or BDNF and PNRb1 treatment concentrations. Online software was used to compute the Spearman’s rank correlation and the two-sided P-value (18). The ordinary scatterplot and scatterplot between the ranks of X and Y were also generated. P<0.05 was considered to indicate a statistically significant difference.

## Results

### BDNF expression is upregulated by PNRb1 in the AD rat model

**Figure 1:**
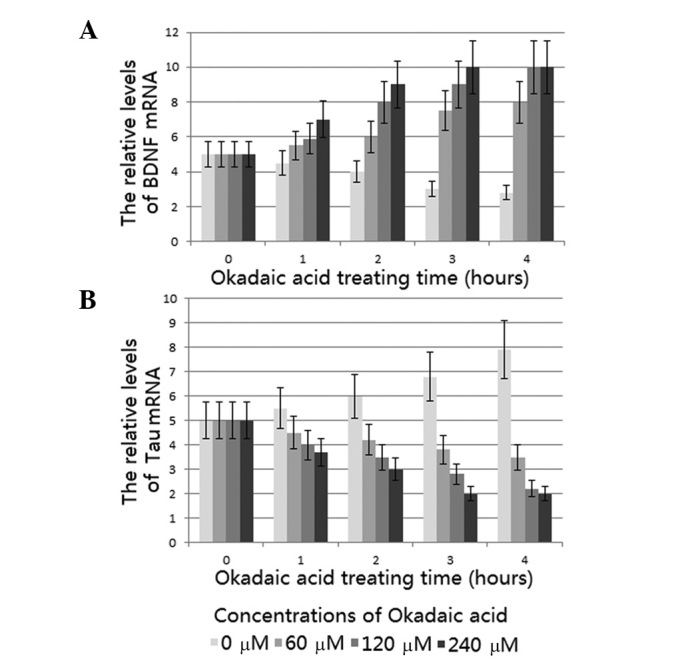
qPCR analysis of the relative levels of brain-derived neurotrophic factor (BDNF) and Tau mRNA, modulated by Panax notoginsenoside Rb1 (PNRb1). Values of the blank control group were taken as one unity to calculate the fold increase. mRNA levels were normalized by glyceraldehyde 3-phosphate dehydrogenase mRNA, whose level did not change during culture with PNRb1. Results are the means of at least three experiments. Values are the mean ± SE.

**Figure 2:**
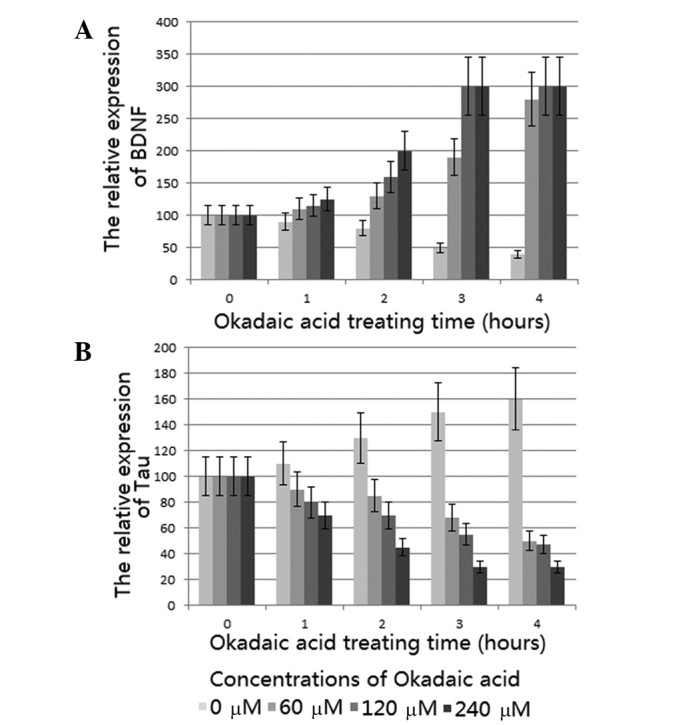
Immunoblot analysis of the relative expression levels of brain-derived neurotrophic factor (BDNF) and phosphorylated Tau protein, modulated by Panax notoginsenoside Rb1 (PNRb1). Values of untreated astrocytes (blank control group) were taken as one unity to calculate the fold increase. Protein levels were normalized by β-tubulin, whose level did not change during culture with PNRb1. Results are the means of at least three experiments. Values are the mean ± SE.

qPCR analysis demonstrated that PNRb1 induced a significant concentration- and time-dependent increase in the BDNF mRNA level compared with that of the model group, which is consistent with the effect of ginsenoside Rg1 (16). The levels of BDNF mRNA were greatest when the tissues were treated with 240 μM PNRb1 for 3 h (Fig. 1A). Corresponding results were also demonstrated in the immunoblot analysis (Fig. 2A); thus, the findings of the qPCR and immunoblot analysis were consistent. BDNF protein expression increased due to the increase in BDNF mRNA.

### Phosphorylated Tau protein is downregulated by PNRb1 in the AD rat model

This study examined the effects of PNRb1 on phosphorylated Tau protein levels in the AD rat model. qPCR analysis showed that PNRb1 induced a significant concentration- and time-dependent reduction of the Tau mRNA level compared with that of the model group, which is consistent with the reported effect of ginsenoside Rg1 (16). Tissues treated with 240 μm PNRb1 for 3 h demonstrated the lowest levels of Tau mRNA (Fig. 1B). Corresponding results were also demonstrated in the immunoblot analysis (Fig. 2B) and therefore, the immunoblot analysis results were consistent with the results from the PCR analysis. Phosphorylated Tau protein expression decreased as the Tau mRNA levels were reduced.

### BDNF and phosphorylated Tau protein are strictly modulated by PNRb1 in the AD rat model

**Figure 3:**
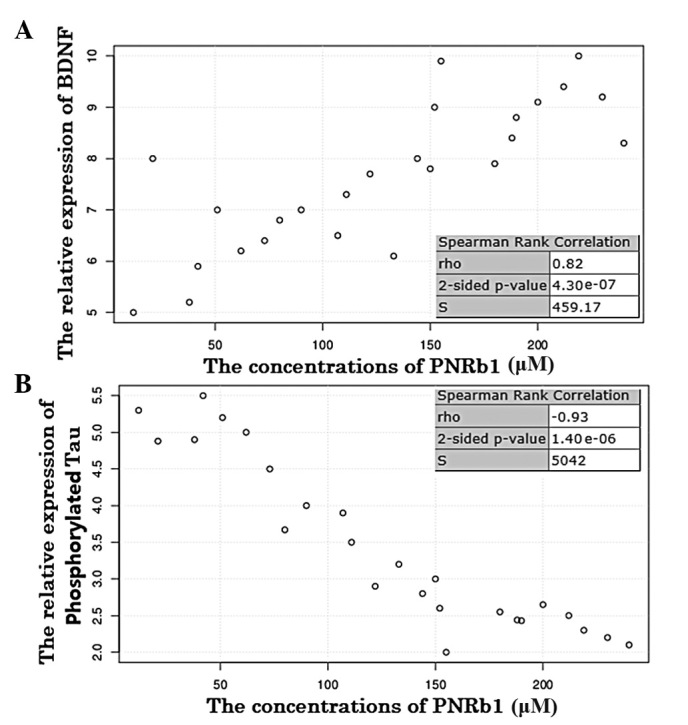
Correlation between the relative expression of (A) brain-derived neurotrophic factor (BDNF) or (B) phosphorylated Tau protein and Panax notoginsenoside (PNRb1) concentration. The AD tissues were treated with 240 μm PNRb1 for 4 h. Statistical analysis was performed using the Spearman’s rank correlation test. Values of rho between 1 and 0.5 indicate a strong positive correlation, while values between −1 and −0.5 imply a strong negative correlation.

The Spearman’s rank correlation coefficient showed that BDNF protein expression and PNRb1 treatment concentrations were significantly and positively correlated in the AD rat model (P<0.001; Fig. 3A). The association between the two variables suggests that BDNF protein expression was upregulated by PNRb1 in the progression of AD.

By contrast, the Spearman’s rank correlation coefficient showed that phosphorylated Tau protein expression and PNRb1 concentration were significantly and inversely correlated in the AD model (P<0.001; Fig. 3B). The inverse correlation between the two variables suggests that phosphorylated Tau protein expression was downregulated by PNRb1 in the progression of AD. Therefore, PNRb1 may be used for the prevention of AD, as it inhibited the phosphorylation of Tau and upregulated the expression of BDNF in the AD model.

## Discussion

An imbalance in the protein kinase and phosphatase system induces Tau protein phosphorylation, resulting in the formation of an abnormally phosphorylated Tau protein (19,20). Cis-trans prolyl isomerization, particularly following phosphorylation, has revealed that cis p-Tau is an early pathogenic conformation that leads to Tau pathology and memory loss in patients with AD (21). Phosphorylated Tau protein participates in the formation of neurofibrillary tangles, resulting in the occurrence of AD. Moreover, the number of neurofibrillary tangles is strongly associated with the degree of dementia in patients with AD (22–24). Okadaic acid, a protein phosphatase-2A inhibitor, is known to enhance Tau phosphorylation, Aβ deposition and neuronal death, which are the pathological hallmarks of AD (25). AD may be detected by investigating the high expression levels of phosphorylated Tau protein (21,26).

The results of the immunoblot analysis demonstrated that phosphorylated Tau protein expression was increased in the AD model group compared with that of the blank control group, which suggests that Tau protein may be an important target during the okadaic acid induction of excessive phosphorylation. Following PNRb1 pretreatment, phosphorylated Tau protein expression was significantly lower than that in the model group. Therefore, PNRb1 was most effective at reducing phosphorylated Tau protein expression.

BDNF is critical in synaptic plasticity and memory processes (27,28). BDNF signaling in the central nuclei of the amygdala and insular cortex, is involved in the consolidation of conditioned taste aversion memory. The differential and spatial-specific roles of BDNF in memory consolidation and reconsolidation suggest that dissociative molecular mechanisms underlie these processes, which may provide novel targets for manipulating newly encoded and reactivated memories without causing universal amnesia (29). It has been proposed that BDNF may protect neurons of the nervous circuitry in patients with AD (30). The BDNF mRNA levels and protein content have been demonstrated to be decreased in the hippocampus and cortex of patients with AD (31). The significant reduction in BDNF expression results in progressive atrophy of the cholinergic system in the basal forebrain and Tau protein phosphorylation in the brains of patients with AD (32), suggesting that BDNF downregulation may be a mechanism of inducing AD.

In the present study, okadaic acid was added to artificial cerebrospinal fluid that was used to incubate rat brain slices. This resulted in diminished BDNF expression in the model group compared with that of the blank control group, which is consistent with decreased BDNF expression in the brains of patients with AD (31). Therefore, it was demonstrated that okadaic acid inhibited BDNF expression. Increased BDNF expression in the brain may improve neuronal survival (33,34), resulting in a delay in or prevention of AD progression.

BDNF has a high molecular weight. If orally administered, exogenous BDNF may be easily damaged by gastric acid. However, with other means of peripheral administration, BDNF is not able to cross the blood brain barrier. Therefore, promoting the production or release of endogenous BDNF may be an effective treatment for patients with AD. In the current study it was demonstrated that PNRb1, in addition to reducing phosphorylated Tau protein expression in the AD model and potentially slowing down the progression of AD, also upregulated BDNF expression and contributed to the production or release of endogenous BDNF.

To the best of our knowledge, the present study was the first to demonstrate the inverse expression pattern between BDNF and phosphorylated Tau, which was modulated by PNRb1. In the progression of AD, BDNF is upregulated by PNRb1 and phosphorylated Tau protein is downregulated by PNRb1, suggesting that PNRb1 may be used for the prevention of AD.
